# Increasing prevalence of overweight and obesity in Yi farmers and migrants from 2007 to 2015 in China: the Yi migrant study

**DOI:** 10.1186/s12889-018-5577-4

**Published:** 2018-05-24

**Authors:** Ye Wang, Li Pan, Shaoping Wan, Huowuli Yi, Fang Yang, Huijing He, Zheng Li, Jia Zhang, Xiaoyang Wang, Zhengping Yong, Guangliang Shan

**Affiliations:** 1 Department of Epidemiology and Statistics, Institute of Basic Medical Sciences, Chinese Academy of Medical Sciences, School of Basic Medicine, Peking Union Medical College, Beijing, China; 2 Sichuan Cancer Hospital and Institute, Sichuan Cancer Center, School of Medicine, University of Electronic Science and Technology of China, Chengdu, China; 3 Puge Center for Disease Control and Prevention, Liangshan, Sichuan, China; 4 Xichang Center for Disease Control and Prevention, Liangshan, Sichuan, China; 5 Sichuan Academy of Medical Sciences and Sichuan Provincial People’s Hospital, Chengdu, Sichuan China

**Keywords:** Overweight, Obesity, Yi people, Rural, Urban, Migrant

## Abstract

**Background:**

With the social development and lifestyle transition, increasing trends of overweight and obesity prevalence are commonly reported worldwide. Data focusing on overweight and obesity trends in rural residents and rural-to-urban migrants in China are limited. This study aims to assess the changes and related factors of overweight and obesity in Yi farmers and migrants in southwest China from 2007 to 2015, and to assess the disparities in prevalence changes.

**Methods:**

Pseudo-panel data was obtained from two cross-sectional studies conducted in Sichuan province, southwest China in 2007 and 2015. Standardized prevalence of overweight and obesity were evaluated by direct method using the 2010 national census population as the standard. Prevalence differences of overweight and obesity were calculated in each group and compared between groups to evaluate the disparity in prevalence changes. Generalized linear mixed model was performed to determine factors related to overweight/obesity.

**Results:**

Standardized prevalence of overweight increased in both groups (from 5.54 to 16.65% in Yi farmers, from 27.91 to 33.61% in Yi migrants). Standardized prevalence of obesity increased only in Yi farmers (from 0.37 to 3.13%). Prevalence difference of overweight in Yi farmers was higher than that in Yi migrants (11.11 vs. 5.70), but prevalence differences of obesity were not significantly different between Yi farmers and migrants.

**Conclusions:**

Prevalence of overweight and obesity in Yi farmers, and prevalence of overweight in Yi migrants increased from 2007 to 2015. Yi farmers were developing overweight at a greater pace than migrants. More attention should be paid to spread health knowledge and encourage healthy lifestyle in Yi people, especially Yi farmers.

**Electronic supplementary material:**

The online version of this article (10.1186/s12889-018-5577-4) contains supplementary material, which is available to authorized users.

## Background

It is well-established that high body mass index (BMI) is a major risk factor for non-communicable diseases (NCDs) including hypertension, diabetes, cardiovascular disease, and cancer [1–3]. Overweight and obesity are fast-growing health care problems worldwide. The increasing prevalence of overweight and obesity has been reported both in developed and developing countries [4]. Globally, obesity prevalence has increased by 2–3 folds in males and females over the past four decades [5]. In China, data obtained from the China Health and Nutrition Survey (CHNS) showed that prevalence of overweight, general obesity and abdominal obesity greatly increased in both genders during the period of 1993–2009 [6].

Migrant studies provide valuable information about effects of transformed social environment and lifestyle behaviors on diseases. Previous studies demonstrated higher risk of obesity in rural-to-urban migrants compared with rural residents [7, 8]. Yi people are one of the ethnic minorities in China residing in remote mountain areas in southwest China. Yi farmers live scattered in mountainous villages, isolated from the urban areas, keeping primitive lifestyle with their own language. Since the 1950s, some Yi farmers began to migrate to urban regions for living and working. Subsequently, rural-to-urban Yi migrants transformed their original lifestyle to similar patterns to local urban residents [9].

Not only for Yi people, China is now undergoing national-scale dramatic modernization and urbanization, with an increasing number of farmers migrating from rural to urban regions. But to our best knowledge, evidences for trends in overweight and obesity prevalence in rural-to-urban migrants and their rural counterparts in China are limited. Fortunately, Yi farmers and migrants provide a perfect opportunity for us to look into this issue. Our previous studies have demonstrated the higher prevalence of hypertension [10], dyslipidemia [11], and metabolic syndrome [12] in Yi migrants than that in Yi farmers. This study aimed to evaluate the changes and related factors of overweight and obesity in Yi farmers and migrants from 2007 to 2015, and to assess the disparities in prevalence changes between the two groups. We hypothesized that the prevalence of overweight and obesity may increase over the eight-year period, and the changes may not be paralleled between Yi farmers and migrants.

## Method

### Study population

The present analysis was based on the Yi Migrant Study. This is a study designed to evaluate cardiovascular risk factors in Yi rural and rural-to-urban migrant population in Liangshan Yi Autonomous Prefecture, Sichuan province, southwest China. Pseudo-panel data was obtained from two independent cross-sectional studies, which were carried out in 2007 and 2015, respectively. Individuals whose parents were both of Yi ethnicity were identified as Yi people. Yi farmers were defined as Yi people who had lived in villages since they were born, and Yi migrants were restricted as Yi people who reported being born in villages and then migrated to urban regions for more than 1 year prior to the surveys. Xichang city (where the municipal of Liangshan Yi Autonomous Prefecture is located), Butuo, Zhaojue, Jinyang, Puge and Xide counties in 2007, Xichang city and Puge county in 2015 were selected as the study fields. Yi farmers were obtained using stratified cluster sampling method from each county. In the first stage, 1–4 townships isolated from the county seat were selected in each county. Then four villages were randomly selected from each township. In the last sampling stage, all Yi farmers aged 20–80 years in selected villages were enrolled. Because of the relative small number of Yi migrants, all of Yi migrants aged 20–80 years found in each county seat and Xichang city were enrolled into the surveys. Written informed consent was obtained from all study participants. Both two surveys were approved by the bioethical committee of Institute of Basic Medical Sciences, Chinese Academy of Medical Sciences, Beijing, China.

### Data collection

In each survey period, standardized questionnaires were used to collect social-demographic characteristics (including sex, age, education, income, and time at first migration) and lifestyles (including smoking, drinking, and physical activity) by face-to-face interviews. The questions were translated by staff who mastered both Chinese and Yi language if the participants spoke Yi language only. Weight was measured in light clothing using an electronic scale (BC-420, TANITA, Japan) with an accuracy of 0.1 kg. Height was measured with barefoot by a fixed stadiometer (TZG-2, HENLONG, China) to the nearest 0.1 cm. The average of three height measurements was recorded. BMI was calculated as weight in kilograms divided by height in meters squared (kg/m^2^). Both questionnaires and anthropometrical measurements were conducted by trained medical staff.

### Definitions

According to WHO criteria [13], overweight was defined as 25 ≤ BMI < 30 kg/m^2^ and obesity was defined as BMI ≥ 30 kg/m^2^. Duration of residence for migrants was calculated based on the survey date and the time when a participant first moved to an urban area from village. Education level was categorized as low (illiteracy or primary school), moderate (middle or high school), and high (college or higher) according to the last educational institution attended. Personal annual income was categorized into three groups in accordance to tertiles. Smoking pack-years were calculated as the average packs of cigarettes smoked per day multiplied by the years of smoking duration. Current smokers were defined as those who smoked at least one cigarette per day for at least 6 months and were classified into three categories: light smokers (0–9 pack-years), moderate smokers (10–19 pack-years), and heavy smokers (20- pack-years). Current drinkers were defined as those who drank at least twice per month with intake of more than 640 ml (one bottle) beer or 100 ml liquor for at least 6 months and were classified into three categories: low-risk drinkers (1-40 g/d for males, 1-20 g/d for females), moderate-risk drinkers (41-60 g/d for males, 21-40 g/d for females), and high-risk drinkers (61- g/d for males, 41- g/d for females) [14]. According to the usual occupational physical activity pattern in the past year, physical activity was classified into three groups: light for sedentary office workers, shop assistants and general housework; moderate for drivers, carpenters and electrician; heavy for manual workers such as building and agricultural laborers [15].

### Statistical analysis

All analyses were conducted using SAS statistical software (Version 9.4; SAS Institute Inc., Cary, NC, USA). Basic characteristics were described as means ± standard deviation (SD) for age and numbers (percentages) for categorical variables according to demographic group (farmer or migrant), sex, and survey period. Student’s t-test and Chi-square test were performed to assess the differences between periods. Covariance analysis for BMI was performed in order to adjust for age. Sex- and age-specific prevalence of overweight and obesity by six 10-year interval age groups (20–29, 30–39, 40–49, 50–59, 60–69, 70–80) were calculated, and standardized prevalence were calculated based on the distributions of 2010 China census population, using the same age categories [16]. Standardized prevalence were assessed by direct method and compared by Z-test. To evaluate the disparities in overweight and obesity changes between Yi farmers and migrants, prevalence differences (PD) were calculated as P_2015_ – P_2007_ and compared. P_2015_ and P_2007_ indicate the standardized prevalence in 2015 and 2007, respectively. The significance of disparity (PD_farmer_ vs. PD_migrant_) was determined by standard error (SE) and 95% confidence interval using the formula: $\sqrt{{\mathrm{SE}}_{\mathrm{farmer}}+{\mathrm{SE}}_{\mathrm{migrant}}}$. In addition, generalized linear mixed model was performed in farmers and migrants separately to determine factors related to overweight/obesity, in which, survey years were inserted in models as independent variable considering as random variable. In the models, overweight and obesity were combined as the dependent variable. A 2-tailed alpha with *P* < 0.05 was considered statistically significant.

## Results

### Basic characteristics

The basic characteristics of the study participants are illustrated in Table 1. Totally, 2295 Yi farmers and 1100 Yi migrants were enrolled in the first survey period, of whom 1071(46.67%) and 656(59.64%) were males. In the second period, 647(34.16%) out of 1894 farmers and 384(33.05%) out of 1162 migrants were males, respectively. Compared with participants in 2007, those in 2015 were older and wealthier. In 2015, a larger proportion of Yi migrants lived in urban regions for more than 30 years. Yi farmers in 2015 had a slight improvement in education level, especially in males. However, a reverse change was observed in Yi migrants. Both in Yi farmers and migrants, the increased proportion of ever smoking and ever drinking were only observed in males but not in females. In 2015, more participants engaged in light physical activities than that in 2007, with an exception for male Yi migrants. The age-adjusted BMI increased in both genders in Yi farmers (from 20.83 kg/m^2^ to 22.30 kg/m^2^ in males, from 21.56 kg/m^2^ to 22.42 kg/m^2^ in females). While in Yi migrants, the increase in BMI was only observed in females, of whom the adjusted BMI increased from 23.46 kg/m^2^ to 24.38 kg/m^2^.

**Table 1:** Characteristics of Yi farmers and migrants, 2007–2015

	Yi Farmer						Yi Migrant					
	Male			Female			Male			Female		
	2007	2015	*P*	2007	2015	*P*	2007	2015	*P*	2007	2015	*P*
N	1071 (46.67)	647 (34.16)	< 0.0001	1224 (53.33)	1247 (65.84)	< 0.0001	656 (59.64)	384 (33.05)	< 0.0001	444 (40.36)	778 (66.95)	< 0.0001
Age (years)	38.63 ± 12.39	46.07 ± 13.31	< 0.0001	40.07 ± 11.59	44.99 ± 12.35	< 0.0001	41.48 ± 11.87	52.02 ± 14.30	< 0.0001	39.68 ± 12.42	47.37 ± 14.17	< 0.0001
Age group			< 0.0001			< 0.0001			< 0.0001			< 0.0001
20–29	287 (26.80)	67 (10.36)		243 (19.85)	115 (9.22)		116 (17.68)	20 (5.21)		100 (22.52)	77 (9.90)	
30–39	292 (27.26)	154 (23.80)		337 (27.53)	330 (26.46)		186 (28.35)	71 (18.49)		136 (30.63)	186 (23.91)	
40–49	258 (24.09)	194 (29.98)		352 (28.76)	409 (32.80)		172 (26.22)	80 (20.83)		112 (25.23)	191 (24.55)	
50–59	180 (16.81)	112 (17.31)		234 (19.12)	212 (17.00)		141 (21.50)	61 (15.89)		59 (13.29)	149 (19.15)	
60–80	54 (5.04)	120 (18.55)		58 (4.74)	181 (14.52)		41 (6.25)	152 (39.58)		37 (8.33)	175 (22.49)	
Duration of residence (years)									< 0.0001			0.0165
1–10	–	–		–	–		146 (22.26)	110 (28.65)		156 (35.13)	276 (35.47)	
11–20	–	–		–	–		192 (29.27)	88 (22.92)		147 (33.11)	233 (29.95)	
21–30	–	–		–	–		170 (25.91)	61 (15.88)		94 (21.17)	138 (17.74)	
31-	–	–		–	–		148 (22.56)	125 (32.55)		47 (10.59)	131 (16.84)	
Education ^a^			< 0.0001			0.0082			< 0.0001			< 0.0001
Low	916(87.32)	471(72.80)		1184(98.59)	1208(96.87)		80(12.56)	99(25.78)		204(47.22)	530(68.21)	
Moderate	130(12.39)	163(25.19)		16(1.33)	31(2.49)		269(42.23)	180(46.88)		148(34.26)	182(23.42)	
High	3(0.29)	13(2.01)		1(0.08)	8(0.64)		288(45.21)	105(27.34)		80(18.52)	65(8.37)	
Income (CNY) ^a^			< 0.0001			< 0.0001			< 0.0001			< 0.0001
0–1000	856(80.91)	151(23.34)		1091(90.39)	393(31.64)		32(4.92)	9(2.35)		41(9.34)	50(6.47)	
1001–5000	182(17.20)	298(46.06)		111(9.20)	630(50.73)		208(31.95)	30(7.81)		153(34.85)	98(12.68)	
5001-	20(1.89)	198(30.60)		5(0.41)	219(17.63)		411(63.13)	345(89.84)		245(55.81)	625(80.85)	
Smoking status ^a^			< 0.0001			< 0.0001			< 0.0001			0.1605
Never smoker	216(20.19)	97(15.02)		1124(91.91)	1171(94.06)		194(29.57)	71(18.49)		415(93.47)	721(92.91)	
Former smoker	21(1.96)	43(6.66)		0(0.00)	12(0.96)		54(8.23)	53(13.80)		4(0.90)	13(1.68)	
0–9 pack-year	275(21.50)	94(14.55)		58(4.74)	25(2.01)		110(16.77)	53(13.80)		14(3.15)	15(1.93)	
10–19 pack-year	239(22.34)	115(17.80)		15(1.23)	15(1.20)		99(15.09)	55(14.32)		7(1.58)	9(1.16)	
20- pack-year	319(29.81)	297(45.98)		26(2.13)	22(1.77)		199(30.34)	152(39.58)		4(0.90)	18(2.32)	
Drinking status			< 0.0001			< 0.0001			< 0.0001			0.2917
Never drinker	497(46.41)	109(16.85)		1132(92.48)	1145(91.82)		172(26.22)	75(19.53)		377(84.91)	663(85.22)	
Ever drinker	26(2.43)	134(20.71)		0(0.00)	20(1.60)		50(7.62)	71(18.49)		7(1.58)	24(3.08)	
Low risk	353(32.96)	264(40.80)		63(5.15)	68(5.45)		308(46.95)	157(40.89)		44(9.91)	74(9.51)	
Moderate risk	40(3.73)	46(7.11)		18(1.47)	5(0.40)		36(5.49)	23(5.99)		7(1.58)	6(0.77)	
High risk	155(14.47)	94(14.53)		11(0.90)	9(0.72)		90(13.72)	58(15.10)		9(2.03)	11(1.41)	
Physical activity ^a^			< 0.0001			< 0.0001			0.5886			< 0.0001
Light	18(1.68)	163(25.19)		20(1.64)	353(28.31)		520(79.39)	303(78.91)		287(64.64)	593(76.22)	
Moderate	32(2.99)	50(7.73)		125(10.22)	64(5.13)		94(14.35)	51(13.28)		101(22.75)	124(15.94)	
Heavy	1020(95.33)	434(67.08)		1078(88.14)	830(66.56)		41(6.26)	30(7.81)		56(12.61)	61(7.84)	
BMI(kg/m^2^)^b^	20.83 ± 0.08	22.30 ± 0.11	< 0.0001	21.56 ± 0.09	22.42 ± 0.09	< 0.0001	24.00 ± 0.15	24.32 ± 0.20	0.1925	23.46 ± 0.19	24.38 ± 0.14	0.0001

Data are presented as mean ± standard deviation or number (percentage);

^a^: Numbers do not sum up to the total due to missing data;

^b^: Lsmeans ± standard error, adjust for age;

Abbreviation: BMI body mass index

### Prevalence of overweight and obesity

**Fig. 1:**
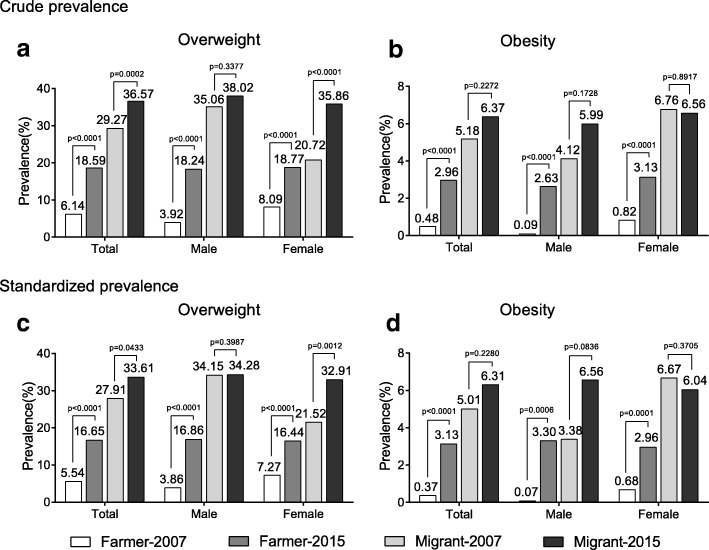
Crude (**a**)(**b**) and standardized (**c**)(**d**) prevalence of overweight (**a**)(**c**) and obesity (**b**)(**d**) in Yi farmers and migrants, 2007-2015. Standardized prevalence of total population were adjusted for age group and sex; standardized prevalence of males and females were adjusted for age group

Figure 1 shows the crude and standardized prevalence of overweight and obesity in the two groups and survey periods. In Yi farmers, from 2007 to 2015, the crude overweight prevalence significantly increased from 6.14 to 18.59% (*P* < 0.0001), and the obesity prevalence increased from 0.48 to 2.96% (*P* < 0.0001). After adjustment for age and sex, the standardized prevalence markedly increased from 5.54 to 16.65%(*P* < 0.0001) for overweight and from 0.37 to 3.13% (*P* < 0.0001) for obesity. The increases in overweight and obesity were observed both in males and females. In Yi migrants, the crude prevalence of overweight increased from 29.27 to 36.57% (*P* = 0.0002), and the standardized prevalence increased from 27.91 to 33.61% (*P* = 0.0433). The changes of both crude and standardized prevalence of overweight were significant in females but not in males. While the changes of obesity prevalence in Yi migrants did not reach the statistical significance, for neither crude nor standardized prevalence.

**Fig. 2:**
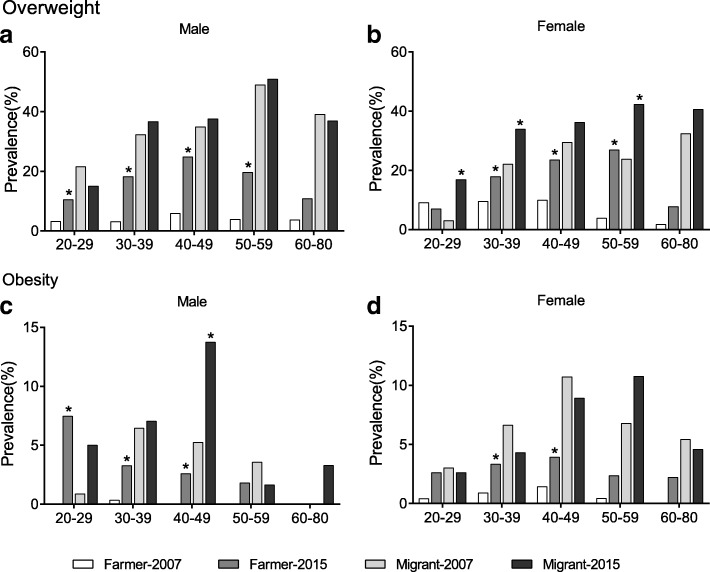
Age specific prevalence of overweight (**a**)(**b**) and obesity (**c**)(**d**) in males (**a**)(**c**) and females (**b**)(**d**) in Yi farmers and migrants, 2007-2015. *: *P*<0.05 for period comparison

The prevalence of overweight and obesity stratified by sex and age group are presented in Fig. 2. The exact number of case and *p*-values are available in Additional file 1: Table S1. Individuals aged 60–69 and 70–80 years were combined because of small sample size. In males, the changes were not consistent between farmers and migrants. In Yi farmers, significant increases were observed through age of 20–59 years for overweight and through 20–49 years for obesity. While in Yi migrants, overweight prevalence were not significantly changed in any age group, and increased obesity prevalence was only observed in the age group of 30–39 years. In females, significant increase in overweight were observed through 30–59 years in Yi farmers, and in adults aged 20–39 and 50–59 years in Yi migrants. For obesity prevalence, the increases were significant only through 30–49 years in female Yi farmers, while no significant change was observed in female Yi migrants in any age group.

### Disparity in prevalence differences

**Fig. 3:**
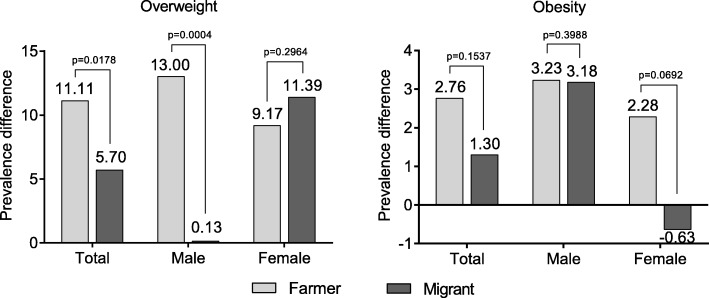
Prevalence difference of overweight and obesity between 2007 and 2015 in Yi farmers and migrants. *Prevalence difference (PD) was calculated as prevalence in 2015 minus prevalence in 2007*

The PDs in Yi farmers and migrants are shown in Fig. 3, which indicate the absolute changes of standardized prevalence of overweight and obesity between the two survey periods. The PD of overweight in Yi farmers was significantly higher than that in Yi migrants (PD_farmer_ = 11.11, PD_migrant_ = 5.70, *P* = 0.0178), especially in males (PD_farmer_ = 13.00, PD_migrant_ = 0.13, *P* = 0.0004). In females, the PDs of overweight in Yi farmers were of no difference with Yi migrants. The PDs of obesity were not significantly different between Yi farmers and migrants, neither in males nor in females.

### Related factors for overweight/obesity

**Fig. 4:**
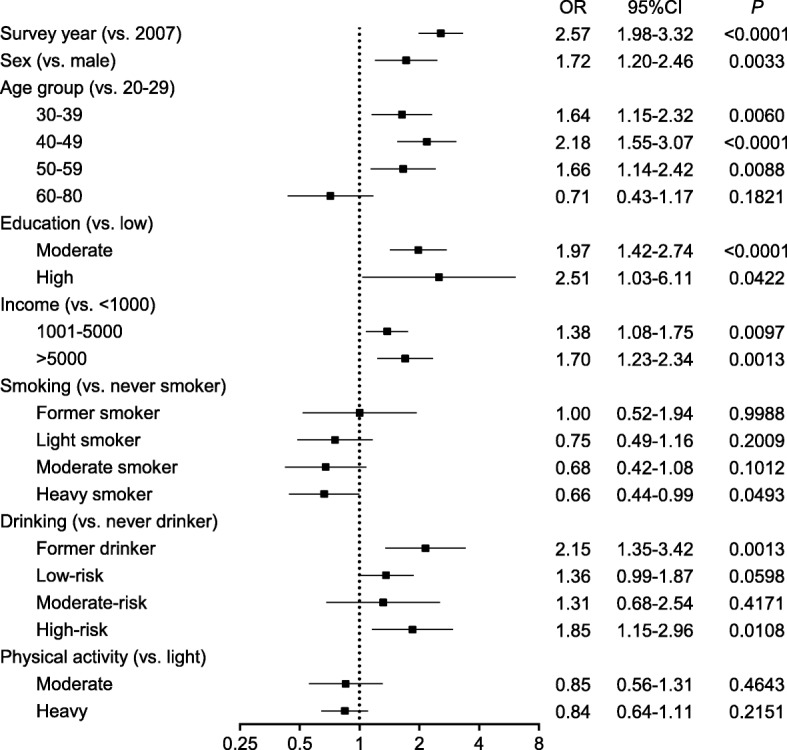
Multiple-adjusted odds ratios for overweight/obesity related factors in Yi farmers. *Overweight and obesity were combined as the dependent variable; Abbreviations: OR: odds ratio, CI: confidence interval*

**Fig. 5:**
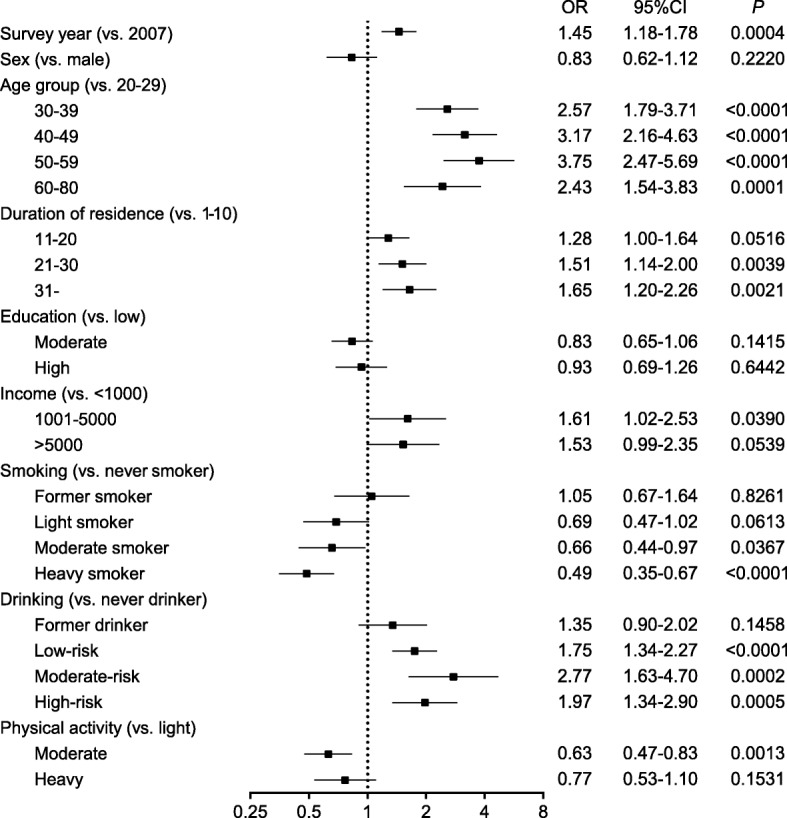
Multiple-adjusted odds ratios for overweight/obesity related factors in Yi migrants, *Overweight and obesity were combined as the dependent variable; Abbreviations: OR: odds ratio, CI: confidence interval*

In multivariable-adjusted analyses, in both Yi farmers and Yi migrants, the risk of overweight/obesity was higher in 2015 than 2007 (Figs. 4 and 5). After adjustment for independent variables, compared to individuals in 2007, the odds ratios of Yi farmers and Yi migrants in 2015 to be overweight/obese were 2.57 (95% CI: 1.98–3.32) and 1.45 (95% CI: 1.18–1.78), respectively. In Yi farmers, female sex, age (30–39, 40–49, 50–59 years), education level (moderate or higher), personal annual income (more than 1000 CNY per year) and drinking status (former drinking and high-risk drinking) were associated significantly with risk of overweight/obesity, whereas heavy smoking was found to be associated with lower risk. In Yi migrants, age (through 30–80 years), duration of urban residence (more than 20 years), personal annual income (1001–5000 CNY per year), and drinking status (current drinking) were associated with higher risk, whereas smoking status (moderate or heavy smoking) and moderate physical activity were associated with lower overweight/obesity risk.

## Discussion

In this study describing the changes of overweight and obesity, according to WHO criteria, we observed increases in standardized prevalence of both overweight and obesity in Yi farmers (from 5.54 to 16.65% for overweight, from 0.37 to 3.13% for obesity, respectively) and increase in standardized prevalence of overweight in Yi migrants (from 27.91 to 33.61%) from 2007 to 2015. In Yi farmers, the increases in overweight and obesity were evident in both gender and in young and middle-aged adults. While in Yi migrants, the changes of overweight were only apparent in females aged 20–39 and 50–59 years.

According to a nationwide survey conducted in 2007–2008, when Working Group on Obesity in China (WGOC) criteria was used (overweight: 24 ≤ BMI < 28 kg/m^2^, obesity: BMI ≥ 28 kg/m^2^) [17], the prevalence of overweight and obesity in Chinese adults aged ≥20 years were 31.4 and 12.2%, respectively [18]. In accordance with the same definition, the corresponding standardized prevalence were 9.2 and 1.2% in Yi farmers contemporaneously. In contrast, the standardized prevalence were relatively parallel in Yi migrants, with the value at 29.8 and 11.3% for overweight and obesity in 2007 (data not shown).

The increases in BMI and overweight/obesity prevalence appear to be a worldwide concern. NCD Risk Factor Collaboration (NCD-RisC) pooled data of 19.2 million people from 200 countries to analyze the global trends of adults BMI from 1975 to 2014. By defining obesity as BMI ≥ 30 kg/m^2^, researchers found that age- standardized prevalence of obesity increased from 3.2 to 10.8% in males, and from 6.4 to 14.9% in females [5]. As to Chinese population, results of China Health and Nutrition Survey (CHNS) showed that during the period of 1993–2009, prevalence of overweight (BMI: 25–27.49 kg/m^2^) increased from 8.0 to 17.1% in males and from 10.7 to 14.4% in females, and prevalence of obesity (BMI ≥27.5 kg/m^2^) increased from 2.9 to 11.4% in men and from 5.0 to 10.1% in women [6].

It is widely accepted that the worldwide overweight and obesity epidemic are essentially attributed to sedentary lifestyles and high-fat energy-dense food intake [19]. The declined levels of physical activity lead to reduction in daily energy expenditure and result in weight gain. In our study, the proportion of Yi people engaging in heavy level of occupational physical activity decreased from 2007 to 2015, except for male Yi migrants. Accordingly, the adjusted BMI and overweight prevalence significantly increased over years in Yi farmers and female Yi migrants but not in male Yi migrants. Similarly, Church et al. [20] proposed that in the United States, the dramatic decreases in work-related physical activity are one of the vital explanations to the obesity epidemic. In addition, it is undeniable that the secular trends can be partially attributed to increasing energy intake. We did not collect data on dietary patterns in the two cross-sectional surveys, but previous studies have demonstrated that in China, the improved food supply and raised food accessibility have contributed to great changes in diet behaviors [21]. Specifically, data from CHNS suggested that the food consumption pattern of Chinese people is shifting towards consumption of high animal-source foods from traditional pattern characterized by intakes of rice and wheat products through the past decades [22]. Although the general nutrition transition along with decreased physical activity likely to play essential roles in the dynamic changes of weight in Yi people, we must note that, Liangshan Yi Autonomous Prefecture, located in southwestern mountainous area of China, is a remote and underdeveloped region, the changes in diet pattern may not be parallel to other developed regions in China. The shift in food consumption pattern and its contribution to the secular trends of obesity deserve future investigation.

In our study, prevalence of overweight increased both in Yi farmers and migrants, but PD between 2015 and 2007 in Yi farmers was greater than migrants. The results reflect the fact that Yi farmers were developing overweight at a more dramatic pace than their rural-to-urban counterparts. Additionally, the increasing prevalence of obesity was significant only in Yi farmers rather than Yi migrants. The rapid increases in overweight and obesity in Yi farmers can be partially explained by the improvement of income. Du et al. [23] found that in China the increased income had larger detrimental effects in low-income group, and the impact of income change over time on lifestyle behaviors varied for different income level. This distinction likely to be caused by disparity in health concept in people with different socioeconomic status. Farmers in low-income level do not have access to excess calories and may be more concerned about the harm of malnutrition than excess weight. Then with the improvement of income, these people get more accessible to high-calorie food but unaware of the health consequence caused by excess energy intake [24]. On the contrary, urban residents tend to be more likely to possess the truth of negative effect that obesity do to health. For another thing, with the shift in mode of agricultural production, more farmers drop manual labor and begin to engage in sedentary lifestyle. Corresponding to our study, the proportion of Yi farmers with light physical activity declined more remarkably than that in Yi migrants. The dynamic trends of overweight and obesity in Yi farmers deserve more public health attention as they may face heavier hypertension and diabetes burden caused by the rising BMI. While the health care expenditure for Yi farmers appears to be a huge burden due to their relative low income.

We observed relative low prevalence of overweight and obesity throughout the age groups in Yi farmers in 2007, especially male Yi farmers, in whom the obesity prevalence was zone except for adults at 30–39 years. Our previous study conducted in the same population demonstrated an extremely low prevalence of hypertension and stable blood pressure levels in all ages [10]. In contrast, with the development of society in 2015, Yi farmers saw elevated prevalence of overweight and obesity in some age groups both in males and females. Our studies identified low cardiovascular disease risk in the remote and underdeveloped population, but the risk increased with the social development and lifestyle shift.

Notably, we found that Yi migrants with longer residence in urban areas had higher risk of overweight/obesity compared with those recently arrived. Similarly, higher BMI and increased prevalence of obesity with longer-term migration have been reported both in transnational [25] and within-country [26] migrants. The positive effects of urban residence length on overweight/obesity risk can be ascribed to acculturation, which means the adoption of obesogenic conditions (i.e. higher energy-dense food and sedentary lifestyle) [27]. Further studies need to be carried out to identify more details in rural-to-urban migrants in China on account of the rapid process of urbanization and rural-to-urban migration [28].

Our study found that smoking was associate with lower risk of overweight/obesity in both Yi farmers and migrants. The data showed that in Yi farmers, heavy smokers (smoking exposure of 20 pack-years or more) had a 33% decreased risk to be overweight/obese when compared to never smokers. In Yi migrants, both moderate or heavy smoking (smoking exposure of 10 pack-years or more) were associated with lower risk. Previous studies identified that cigarette smoking could lead to weight loss by increasing the metabolic rate, decreasing metabolic efficiency or decreasing caloric absorption (reduction in appetite by nicotine) [29]. However, considering the adverse effects of cigarette on health, it should not be encouraged to control body weight by adopting smoking.

The relationship between alcohol drinking and overweight/obesity was controversial. Several previous studies reported nonlinear relationship between alcohol drinking and BMI, with light-to-moderate drinking being negatively associated with adiposity indicators [30]. In our study, we identified current drinking as a risk factor of overweight/obesity. In Yi farmers, high-risk drinkers (males who drink more than 60 g alcohol per day, females who drink more than 40 g alcohol per day) had 85% increased risk than never drinkers. Besides, the association was stronger in Yi migrants. Even current drinkers with low- or moderate-risk amount had significantly higher risk. It is recognized that increased energy intake with alcohol consumption can certainly promote a positive energy balance and ultimately weight gain [31].

One of the strengths of our study lies in the collection of data from two population with same genetic background while in diverse living environment in two periods. The sample allows us to conduct comprehensive study in the changes of overweight/obesity as well as other non-communicable diseases, and to assess the association with changed environmental factors. Although our study was based on two independent cross-sectional surveys rather than a cohort, the results were reliable and comparable because: Firstly, the same sampling method and excluding criterions were used to obtain representative samples in the two surveys. Second, interviews and anthropometric measurements were carried out by well-trained personnel following standard method in the two surveys. Several limitations in our study deserve consideration. Firstly, due to the cross-sectional nature of our study, causality cannot be established. Secondly, we only used BMI to define overweight and obesity, which cannot distinguish fat and lean tissue or the body fat distribution [32]. But as a parameter that can be easily calculated by body weight and height, BMI is widely used in epidemiological surveys and is valuable for international comparisons [33]. In addition, in terms of physical activity, leisure time physical exercises or transportation were not taken into consideration. However, it is recognized that the work-related physical activity primarily determines the daily energy expenditure by activity [20]. Furthermore, the sample size limited the power to determine the changes in prevalence, particularly in subgroups stratified by sex and age group. Despite of the limitations mentioned above, our study did provide a relevant evidence for overweight and obesity prevention.

## Conclusions

In conclusion, our study suggests increasing prevalence of overweight and obesity in Yi farmers, and increase in overweight in Yi migrants over the eight-year period. In spite of the relative low prevalence, Yi farmers were developing overweight at faster pace than Yi migrants. In order to contain the secular trends of overweight and obesity and to prevent obesity-related diseases, at the same time as socio-economic development, health knowledge should be disseminated and healthy life styles should be adhered, especially in Yi farmers.

## Additional file

Additional file 1: **Table S1.** Sex and age specific prevalence of overweight and obesity in Yi farmers and migrants, 2007–2015. (DOCX 19 kb)

## Abbreviations

BMI: Body mass index
CHNS: China Health and Nutrition Survey
CI: Confidence interval
NCDs: Non-communicable diseases
OR: Odds ratios
PD: Prevalence difference
WGOC: Working Group on Obesity in China
