# Supplementary material for: Increasing prevalence of overweight and obesity in Yi farmers and migrants from 2007 to 2015 in China: the Yi migrant study
Source: BMC Public Health. 2018 May 24;18:659. doi: 10.1186/s12889-018-5577-4 (PMC5968478; doi:10.1186/s12889-018-5577-4)
Supplement: Supplementary file 1 — Table S1. Sex and age specific prevalence of overweight and obesity in Yi farmers and migrants, 2007–2015. (DOCX 19 kb) [file 12889_2018_5577_MOESM1_ESM.docx]

Additional file 1: Table S1. Sex and age specific prevalence of overweight and obesity in Yi farmers and migrants, 2007-2015

|  |  | Farmer | | |  | Migrant | | |
| --- | --- | --- | --- | --- | --- | --- | --- | --- |
|  |  | 2007 | 2015 | *P* |  | 2007 | 2015 | *P* |
|  |  | Overweight | | | | | | |
| Male | |  |  |  |  |  |  |  |
|  | 20-29 | 3.14 (9/287) | 10.45 (7/67) | **0.0234** |  | 21.55 (25/116) | 15.00 (3/20) | 0.7115 |
|  | 30-39 | 3.08 (9/292) | 18.18 (28/154) | **<0.0001** |  | 32.26 (60/186) | 36.62 (26/71) | 0.5076 |
|  | 40-49 | 5.81 (15/258) | 24.74 (48/194) | **<0.0001** |  | 34.88 (60/172) | 37.50 (30/80) | 0.6866 |
|  | 50-59 | 3.89 (7/180) | 19.64 (22/112) | **<0.0001** |  | 48.94 (69/141) | 50.82 (31/61) | 0.8058 |
|  | 60-80 | 3.7 (2/54) | 10.83 (13/120) |  |  | 39.02 (16/41) | 36.84 (56/152) |  |
| Female | |  |  |  |  |  |  |  |
|  | 20-29 | 9.05 (22/243) | 6.96 (8/115) | 0.5037 |  | 3.00 (3/100) | 16.88 (13/77) | **0.0014** |
|  | 30-39 | 9.50 (32/337) | 17.88 (59/330) | **0.0016** |  | 22.06 (30/136) | 33.87 (63/186) | **0.0209** |
|  | 40-49 | 9.94 (35/352) | 23.47 (96/409) | **<0.0001** |  | 29.46 (33/112) | 36.13 (69/191) | 0.2362 |
|  | 50-59 | 3.85 (9/234) | 26.89 (57/212) | **<0.0001** |  | 23.73 (14/59) | 42.28 (63/149) | **0.0125** |
|  | 60-80 | 1.72 (1/58) | 7.73 (14/181) |  |  | 32.43 (12/37) | 40.57 (71/175) |  |
|  |  | Obesity | | | | | | |
| Male | |  |  |  |  |  |  |  |
|  | 20-29 | 0.00 (0/287) | 7.46 (5/67) | **0.0002** |  | 0.86 (1/116) | 5.00 (1/20) | 0.2734 |
|  | 30-39 | 0.34 (1/292) | 3.25 (5/154) | **0.0358** |  | 6.45 (12/186) | 7.04 (5/71) | 0.9999 |
|  | 40-49 | 0.00 (0/258) | 2.58 (5/194) | **0.0325** |  | 5.23 (9/172) | 13.75 (11/80) | **0.0199** |
|  | 50-59 | 0.00 (0/180) | 1.79 (2/112) | 0.1463 |  | 3.55 (5/141) | 1.64 (1/61) | 0.7783 |
|  | 60-80 | 0 (0/54) | 0 (0/120) |  |  | 0 (0/41) | 3.29 (5/152) |  |
| Female | |  |  |  |  |  |  |  |
|  | 20-29 | 0.41 (1/243) | 2.61 (3/115) | 0.1907 |  | 3.00 (3/100) | 2.60 (2/77) | 0.9999 |
|  | 30-39 | 0.89 (3/337) | 3.33 (11/330) | **0.0278** |  | 6.62 (9/136) | 4.30 (8/186) | 0.3585 |
|  | 40-49 | 1.42 (5/352) | 3.91 (16/409) | **0.0364** |  | 10.71 (12/112) | 8.90 (17/191) | 0.6044 |
|  | 50-59 | 0.43 (1/234) | 2.36 (5/212) | 0.1750 |  | 6.78 (4/59) | 10.74 (16/149) | 0.3827 |
|  | 60-80 | 0 (0/58) | 2.21 (4/181) |  |  | 5.41 (2/37) | 4.57 (8/175) |  |

Overweight: 25 ≤ BMI < 30 kg/m^2^

Obesity: BMI ≥ 30 kg/m^2^
